# Comparative UAV and Field Phenotyping to Assess Yield and Nitrogen Use Efficiency in Hybrid and Conventional Barley

**DOI:** 10.3389/fpls.2017.01733

**Published:** 2017-10-10

**Authors:** Shawn C. Kefauver, Rubén Vicente, Omar Vergara-Díaz, Jose A. Fernandez-Gallego, Samir Kerfal, Antonio Lopez, James P. E. Melichar, María D. Serret Molins, José L. Araus

**Affiliations:** ^1^Integrative Crop Ecophysiology Group, Department of Evolutionary Biology, Ecology and Environmental Sciences, University of Barcelona, Barcelona, Spain; ^2^Syngenta España, Madrid, Spain; ^3^Syngenta United Kingdom, Cambridge, United Kingdom

**Keywords:** hybrid barley, *Hordeum vulgare*, nitrogen, vegetation index, UAV, RGB, multispectral, thermal

## Abstract

With the commercialization and increasing availability of Unmanned Aerial Vehicles (UAVs) multiple rotor copters have expanded rapidly in plant phenotyping studies with their ability to provide clear, high resolution images. As such, the traditional bottleneck of plant phenotyping has shifted from data collection to data processing. Fortunately, the necessarily controlled and repetitive design of plant phenotyping allows for the development of semi-automatic computer processing tools that may sufficiently reduce the time spent in data extraction. Here we present a comparison of UAV and field based high throughput plant phenotyping (HTPP) using the free, open-source image analysis software FIJI (Fiji is just ImageJ) using RGB (conventional digital cameras), multispectral and thermal aerial imagery in combination with a matching suite of ground sensors in a study of two hybrids and one conventional barely variety with ten different nitrogen treatments, combining different fertilization levels and application schedules. A detailed correlation network for physiological traits and exploration of the data comparing between treatments and varieties provided insights into crop performance under different management scenarios. Multivariate regression models explained 77.8, 71.6, and 82.7% of the variance in yield from aerial, ground, and combined data sets, respectively.

## Introduction

Global nitrogen fertilizer use is expected to exceed 200 million tons in the next year and continue to increase at 1.8% per year (FAO et al., 2015). The estimated increase in fertilizer use by 22.4% globally over the last 10 years (2004–2014) differs from an increase of 14.3% at the European level, where there is stronger and growing presence of precision agriculture management practices (Food and Agriculture Organization of the United Nations, 2017), but still demonstrates a growing demand for increased yields and an upwards trend in intensity of agricultural practices. Strikingly, this increase in fertilizer application only corresponded to barley (*Hordeum vulgare*) yield increases of 9.3 and 10.6% at the global and European scales, respectively. For barley, the fourth global grain in terms of production (FAO et al., 2015), this represents only 5.7 and 3.3% increases in nitrogen use efficiency (NUE) over a period of 10 years, approximately half of which is attributed to improved management practices (Raun and Johnson, 1999). Given global barley production at 144.6 million tons annually, improvements in NUE represent significant savings that may enable production to meet future demand and increase profits (Raun and Johnson, 1999). Barley NUE is estimated to have been improved by nearly 26% over the past 100 years of active breeding in developed countries, with little signs of impediment, but active phenotyping toward NUE has declined since the Green Revolution in favor or maximum yield under optimum conditions (Raun and Johnson, 1999; Rajala et al., 2017). With previously estimated NUEs of 42 and 29% between developed and developing countries, much of this may be attributed to management practices such as variable rate N applications based on precision agriculture technology (Anbessa and Juskiw, 2012); however, improved NUE related to cultivars and hybrid selection has long been focused on top yield with N fertilizer intentionally removed from the study to focus on other selection criteria (Raun and Johnson, 1999; Hirel et al., 2007). Though some previous studies have covered NUE and yield differences between two-row and six-row barley varieties (Le Gouis, 1992; Papastylianou, 1995; Le Gouis et al., 1999; Frégeau-Reid et al., 2001; del Moral et al., 2003; Arisnabarreta and Miralles, 2008), hybrid barley may represent an alternative not only in terms of higher yield but also of improved growth and NUE (Gorny and Sodkiewicz, 2001; Kostadinova et al., 2016); however, to date there are no studies that we know of aimed at proving the effectiveness of remote sensing techniques as phenotyping tools for assessing the higher performance of hybrid barley in terms of growth, grain yield and NUE.

New techniques in high throughput plant phenotyping (HTPP) can provide key assistance in gathering data in support of assessing key crop physiological traits for breeding selection programs, including quantifying the physiological condition of crops, prediction of yield pre-harvest, and the heritability of traits such as increased resources use efficiencies (Furbank and Tester, 2011; Cabrera-Bosquet et al., 2012; Fiorani and Schurr, 2013; Araus and Cairns, 2014; Araus et al., 2014). In the past these techniques, applied to field phenotyping, have focused largely on the improvement in efficiency in time and cost of gathering the most important data, which remained still fairly time consuming due to the need for numerous replicates and varietal comparisons in traditional phenotyping studies. Technological advancements have been more focused on the use of robotics and automated processing of replicates and crosses in controlled laboratory conditions (Hawkesford and Lorence, 2017) and with large, expensive field machinery (Virlet et al., 2017); however, recent technological advancements in Unmanned Aerial Vehicles, UAVs, toward more stable and affordable research platforms and sensor engineering (lighter sensors with higher spatial and spectral resolution) have brought the capacity for increasing automation in a wider range of field HTPP conditions and budgets (Kefauver et al., 2015; Zhou et al., 2015; Vergara-Diaz et al., 2016).

In traditional plant breeding and phenotyping studies, the measurement of objective traits relevant for plant breeding needs to be acquired as efficiently possible to achieve statistical confidence (Montes et al., 2007; White et al., 2012; Fiorani and Schurr, 2013; Araus and Cairns, 2014). To this aim, the very high resolution and inherent color calibration of commercial digital RGB (Red, Green and Blue, or visible light) may provide fast quality image-based data acquisition (Fiorani et al., 2012; Akkaynak et al., 2014). Through not scientifically designed sensors, commercial RGB cameras include rigorous factory color calibration that enables their use for extensive scientific capacity, considering that it is in the visible where plant pigments related to photosynthesis capture light—color quality is inherently tied to photosynthesis. From this a specific suite of vegetation indices (VIs) were developed by Casadesús et al. (2007); Casadesús and Villegas (2014) to take advantage of transformations from the RGB to the CIElab, CIEluv, and HSB color spaces that can in effect remove artifacts of illumination variation and more accurately quantify the relative abundance of different pigments in plants at very detailed spatial resolutions. In a sense similar to traditional multispectral indices, Hunt et al. (2011, 2013) used RGB cameras to calculate normalized and area-based indices based on the broad band reflectance of each band, namely the Triangular Greenness Index and the Normalized Green Red Difference Index.

In this study, we compared the technical capacity of field- and UAV-based RGB and multispectral image analyses to differentiate the nitrogen related performance between two barley hybrids and a commercial line of barley that is widely cultivated in the region where the experiment was performed. While the emphasis of our study is in the performance of affordable remote sensing approaches (derived from RGB images), for comparison to these novel low-cost commercial RGB camera image analyses, a commercial scientific multispectral sensor was also mounted on the same UAV in order to compare the quality and cost-effectiveness of the field data quantification with each sensor type. The multispectral sensor payload included a camera array of 11 separate spectral bands with an upwards pointing incident light sensor for simultaneous calibration to reflectance and a thermal camera. We calculated from the multispectral camera a suite of scientific spectral reflectance indices for the purposes of comparing the nitrogen use efficiency and yield component estimating capabilities of different combinations of high spatial (16 megapixel), low spectral resolution (RGB) sensors with low spatial resolution (1.3 megapixel) and moderate multispectral resolution (11 band) scientific sensors from a UAV HTPP. To further explore the benefits of high spatial resolution, the same RGB analyses conducted from the UAV data taken at a 50 m altitude was further compared with data acquired from an equal resolution commercial RGB digital camera at approximately 1 m above the canopy of each study plot.

## Materials and methods

### Plant material and experimental setup

**Figure 1:**
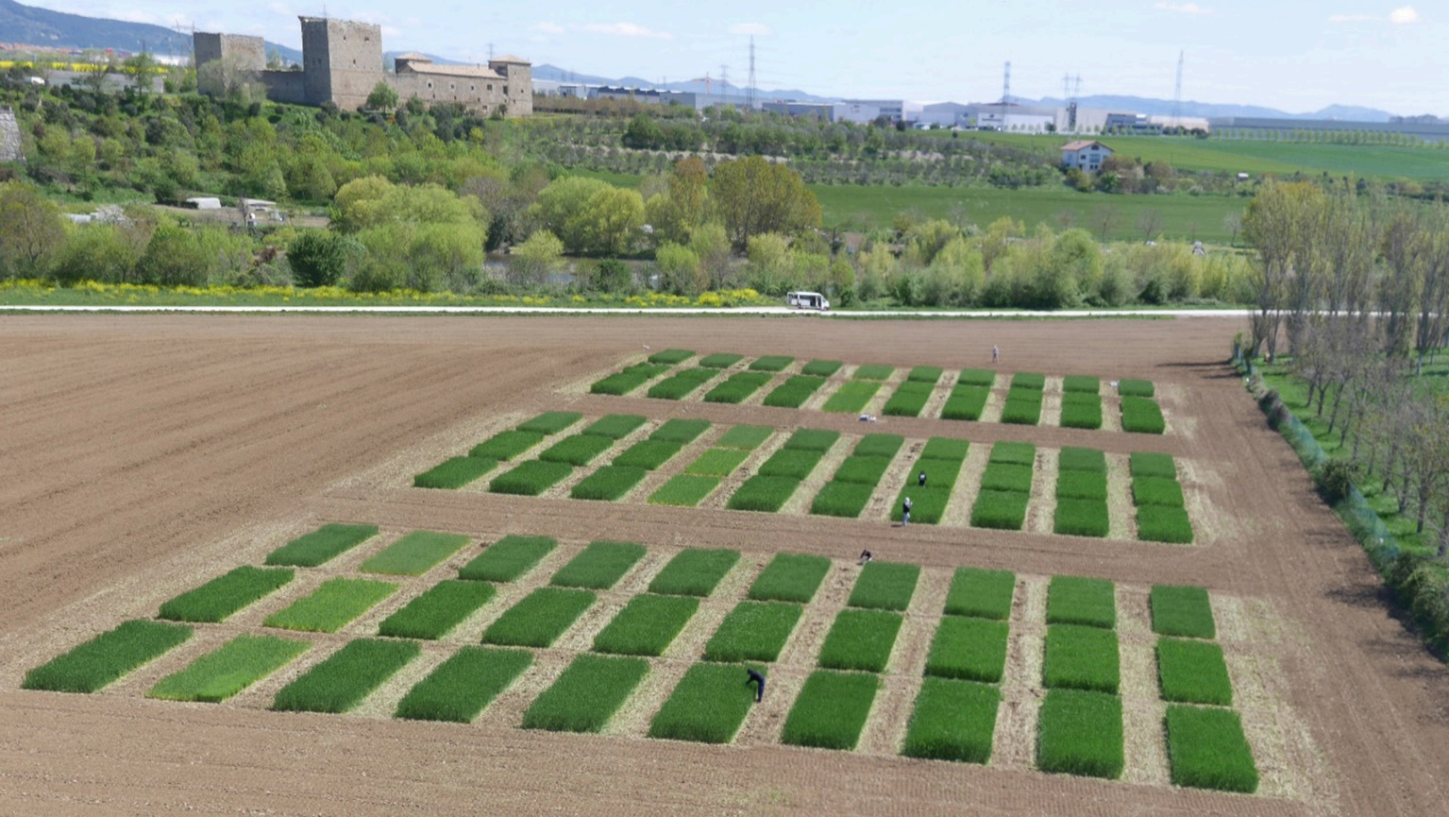
Field experiment at Arazuri Station in Navarra (Spain) during the crop season of 2015/2016.

The field trial was carried out during the 2015/2016 crop season in the Arazuri Station of the Institute of Agrifood Technologies and Infrastructures of Navarra (INTIA), located in Navarra, Spain (42° 48′ N, 1° 43′ W, 396 m a.s.l.). The climate conditions of the region represents a transition from Mediterranean to Atlantic climate, with a mean, maximum and minimum daily air temperature of 12.9°, 18.8°, and 7.6°C, respectively, average relative humidity of 75.2%, and annual precipitation of 854 mm during 2015. Three barley (*H. vulgare* L.) genotypes were used in this study, one conventional two-row cultivar (Meseta), and two six-row hybrids (Jallon and Smooth). Meseta is a winter barley variety widely cultivated in Spain due to its high yield potential in most areas. Jallon and Smooth are two winter barley hybrids released by Syngenta S.A.U. using the Hyvido™ technology. Barley seeds were planted in plots of 12 m^2^ (10 × 1.2 m) with 8 lines per plot separated by 15 cm at the recommended sowing rate for each variety on November 16, 2015. Initially, 10 different N fertilization regimens were established in the trial, differentiated in application dates and doses (Table 1). A mix of urea and ammonium sulfate was applied on January 25, 2016 in the first fertilizer applications, and urea was applied in all of the subsequent fertilizer applications. The experimental design was performed in randomized blocks with three replicates per genotype and N treatment combination, with a total of 90 plots (Figure 1). Weeds, insect pests and diseases were controlled by the application of the recommended pesticides for the location, including *Axial Pro (Syngenta)* and *Trimmer* (tribenuron-methyl, Conquest) herbicides at the recommended doses in one single application on April 26, 2016.

**Table 1:** N treatments and application dates supplied during the life cycle of barley plants from three genotypes (Meseta, Jallon and Smooth).

**References**	**N supply**		**Pre-sowing**		**January (emergence)**		**February (tillering)**		**April (booting)**
N0	0	=	0	+	0	+	0	+	0
N130a	130	=	0	+	65	+	0	+	65
N130b	130	=	40	+	50	+	0	+	40
N150a	150	=	0	+	65	+	0	+	85
N150b	150	=	40	+	0	+	110	+	0
N150c	150	=	40	+	50	+	0	+	60
N170a	170	=	0	+	65	+	0	+	105
N170b	170	=	0	+	85	+	0	+	85
N170c	170	=	40	+	0	+	130	+	0
N170d	170	=	40	+	50	+	0	+	80

*Values are expressed in kg ha^−1^*.

Direct measurement parameters such as vegetation indices (VIs) from ground, as well as image data acquisition with the UAV for the calculation of canopy temperature and other multispectral VIs were recorded at the growth stage of booting in all plots. All the measurements were performed during the morning and early afternoon between 10:00 and 15:00 (except the canopy temperature at afternoon). Agronomic traits were determined at physiological maturity for every plot.

### Agronomical traits and vegetation indices (VIs) from ground

Agronomic traits such as thousand grain weight (TGW), number of grains per area (NG) and grain yield (GY) were determined at harvest. The harvest was done with a micro-plot combine-harvester equipped with an automatic weighing system. Additionally, the agronomical NUE (aNUE) and the nitrogen partial factor productivity (NPFP) were calculated according to (Lü et al., 2012):

$$\begin{array}{lll} aNUE\;\left(g\;grain/g\;N_{supply}\right) & = & \left(GY-GY\;for\;zero\;N\;plot\right)/N_{supply}\\ NPFP\;\left(g\;grain/g\;N_{supply}\right) & = & \;GY\;for\;N\;treated\;plot/N_{supply} \end{array}$$

Normalized Difference Vegetation Index (NDVI) was evaluated with an active sensor hand-held portable spectroradiometer (GreenSeeker, NTech Industries, Ukiah, CA, USA) by passing the sensor in the middle of each plot at a constant height of 0.5 m above and perpendicular to the canopy. This index is calculated from the red and near infrared (NIR) light reflected by vegetation using the following equation: (NIR − Red) / (NIR + Red). Chlorophyll content was measured in situ using a portable chlorophyll meter SPAD-502 (Minolta, Tokyo, Japan). The measurements were carried out in the central segment of the leaf lamina, using the flag leaves of 10 plants per plot selected randomly and averaged.

For each plot, one RGB picture was taken by holding the camera at 0.8-1.0 m above the canopy in a zenithal plane and focusing near the center of the plot. A camera Olympus E-M10 (Olympus Corporation, Tokyo, Japan) was used with a focal length of 14 mm, shutter speed of 1/250, no flash and the aperture in automatic. The pictures were saved in JPEG format with a resolution of 4608 × 3072 pixels. These were analyzed with the open source BreedPix 0.2 software (Casadesús et al., 2007) for the calculation of RGB indices in the canopy based on different properties of color. In this study we used five color components of potential interest as VIs: hue, saturation, intensity, lightness, a^*^, b^*^, u^*^, v^*^, and the relative green area (GA) and greener area (GGA) (Casadesús et al., 2007; Vergara-Diaz et al., 2015, 2016). Hue belongs to the HSI color space (Hue, Saturation, Intensity), where Hue refers to the color tint, while Saturation follows as the amount of tint (ranging from an intense color to white) and Intensity is the overall albedo or brightness of the image (ranging from black to white). With regards to the CIE-Lab and CIE-Luv color spaces (recommended by the International Commission on Illumination (CIE) for improved color chromaticity compared to HIS color space), L represents Lightness in both CIE-Lab and CIE-Luv and is similar to Intensity or overall albedo from the HIS color space, whereas a^*^ and u^*^ represent the red-green spectrum of chromaticity and b^*^ and v^*^ represent yellow-blue color spectrum. Finally, GA and GGA indicate the green biomass in the picture calculated using the Hue channel of HIS color space as it is detailed in Casadesús et al. (2007), with the latter excluding yellowish pixels that correspond to senescent leaves. Additionally, crop senescence index (CSI), which effectively provides a scaled ration between yellow and green vegetation pixels in the image, was calculated in agreement with Zaman-Allah et al. (2015) as follows:

$$\begin{array}{l} CSI=100\times \left(GA-GGA\right)/GA. \end{array}$$

### Canopy temperature and vegetation indices from aerial images

All UAV aerial images were acquired on April 27, 2016 in clear sky conditions using a Mikrokopter Oktokopter 6S12 XL eight rotor UAV (HiSystems GmbH, Moomerland, Germany) flown manually at a steady velocity of approximately 5 m/s and a pressure altimeter stabilized a.g.l. altitude of 50 m. Image acquisition for each sensor was programmed for continuous capture for the duration of each flight with image acquisition rates of 2 s, 20/s and 5 s for the RGB, thermal and multispectral cameras, respectively, representing the maximum recommended image acquisition speed for each camera sensor system. All cameras were mounted on the same MK HiSight SLR2 camera platform with an active two servo axis gimbal to correct for UAV pitch and roll movements during flight. Different payload configurations allowed for the four image datasets to be gathered in three flights. The first included digital RGB and thermal cameras, the second flying only with the multispectral camera array, and the third flight with the thermal camera alone. Nadir image acquisition alignment accuracy was assessed and proper gimbal function was tested prior to each flight.

The RGB images were recorded starting at 11:47 am using a 16 megapixel micro 4/3 sensor Panasonic GX7 digital camera (Panasonic Corporation, Osaka, Japan) with a 20 mm “pancake” lens set at automatic focus with a fixed exposure time and aperture and programmed to record images continuously at intervals of 2 s for the duration of the flight. RGB images were imported and filtered to include only nadir images from each flight line over of the study site at the 50 m a.g.l. before further processing into orthomosaics. The estimated resulting pixel resolution of the RGB images was calculated at a 10 mm ground spatial resolution per pixel.

Canopy temperature was measured at morning (T_mor_, 13:01 pm) and afternoon (T_aft_, 16:51 pm) using a FLIR Tau2 640 (FLIR Systems, Nashua, NH, USA) thermal camera with a VOx uncooled microbolometer equipped with a TEAX ThermalCapture module (TEAX Technology, Wilnsdorf, Germany) for recording of full resolution thermal video (640 x 520 pixels at 20 frames per second) with an estimated ground spatial resolution of 54 mm per pixel. The thermal images were first exported using the TEAX ThermoViewer v1.3.12 in raw 16 bit TIFF format as Kelvin ^*^ 10,000 and converted to 32 bit temperature in Celsius using a custom batch processing macro function in FIJI (Schindelin et al., 2012).

The multispectral data from the Tetracam (Tetracam, Inc., Gainesville, FL) mini MCA (Multiple Camera Array) 11 plus Incident Light Sensor (ILS) camera includes 12 individual image sensors with filters of center wavelengths and full-width half-max bandwidths of 450 ± 40, 550 ± 10, 570 ± 10, 670 ± 10, 700 ± 10, 720 ± 10, 780 ± 10, 780 ± 10, 840 ± 10, 860 ± 10, 900 ± 20, 950 ± 40 nm, and one sensor dedicated to real-time reflectance calibration (ILS) with a 30 cm fiber optic cable connected to an upwards looking box with a light diffusion plate containing 11 matching filters corresponding exactly to the 11 downwards looking sensor filters. Data acquisition was programmed for every 5 s for the duration of the flight at 50 m a.g.l., resulting in 12-band 8 bit TIFF images at 1,280 × 1,024 pixels with a ground spatial resolution estimated at 27 mm spatial resolution per pixel. For processing, each of the resulting 12 separate images from each sensor were first aligned to correct for parallax according to the Pixel Wrench II version 1.2.2.2 Field of View (FOV) Optical Calculator and calibrated to reflectance using the PixelWrench II version 1.2.2.2 Index Tools MCA and ILS functions in a batch function with the provided factory calibration parameters.

The preprocessed exported images from each sensor were then combined into orthomosaics to correct for terrain and UAV flight movement by camera type for each flight using Agisoft Photoscan Professional (Agisoft LLC, St. Petersburg, Russia). Each orthomosaics image was then cut to create mini-raster images for each individual study plot using FIJI and then batch processed using custom macro functions for index calculations and simultaneous data export for each sensor, including one orthomosaic for the RGB and multispectral sensors, and morning and afternoon orthomosaics from the thermal camera.

The same RGB VIs that were calculated as described in section Agronomical traits and vegetation indices (VIs) from ground were also calculated using RGB aerial images acquired using from the UAV using the exported plot level images in the same manner as the RGB images captured in the field. The thermal data average temperature and standard deviation were exported per plot using a custom macro developed in FIJI (Schindelin et al., 2012). A suite of multispectral indices were calculated from the 11 multispectral reflectance bands of the Tetracam mini MCA 11+ILS as described herewith, where R indicates reflectance and the subscripts indicate wavelengths in nm, including the NDVI calculated as Rouse et al. (1974),

$$\begin{array}{l} NDVI=\left(R_{840}-R_{670}\right)/\left(R_{840}+R_{670}\right); \end{array}$$

the Photochemical Reflectance Index (PRI) (Gamon et al., 1992),

$$\begin{array}{l} PRI=\left(R_{550}-R_{570}\right)/\left(R_{550}+R_{570}\right); \end{array}$$

Soil Adjusted Vegetation Index (SAVI) (Huete, 1988),

$$\begin{array}{l} SAVI=\left(R_{840}-R_{670}\right)/\left(R_{840}+R_{670}+L\right)\times \left(1+L\right), \end{array}$$

where L is a canopy background adjustment factor with an optimal value of L = 0.5;

the Modified Chlorophyll Absorption Ratio Index (MCARI) (Daughtry et al., 2000),

$$\begin{array}{l} MCARI=\left[\left(R_{700}-R_{670}\right)-0.2\times \left(R_{700}-R_{550}\right)\right]\times \left(R_{700}/R_{670}\right); \end{array}$$

Water Band Index (WBI) (Peñuelas et al., 1993),

$$\begin{array}{l} WBI=\left(R_{900}/R_{950}\right); \end{array}$$

Renormalized Difference Vegetation Index (RDVI) (Roujean and Breon, 1995),

$$\begin{array}{l} RDVI=\left(R_{840}-R_{670}\right)/{\left(R_{840}+R_{670}\right)}^{1/2}; \end{array}$$

Enhanced Vegetation Index (EVI) (Huete et al., 2002),

$$\begin{array}{l} EVI=2.5\times \left(\left(R_{840}-R_{670}\right)/\left(R_{840}+6\times R_{670}-7.5\times R_{450}+1\right)\right); \end{array}$$

Anthocyanin Reflectance Index 2 (ARI2) (Gitelson et al., 2001),

$$\begin{array}{l} ARI2=R_{840}\times \left[\left(1/R_{550}\right)-\left(1/R_{700}\right)\right]; \end{array}$$

Carotenoid Reflectance Index 2 (CRI2) (Gitelson et al., 2002),

$$\begin{array}{l} CRI2=\left(1/R_{550}\right)-\left(1/R_{700}\right); \end{array}$$

Transformed Chlorophyll Absorption Ratio Index (TCARI) (Haboudane et al., 2002),

$$\begin{array}{l} TCARI=3\times \left[\left(R_{700}-R_{670}\right)-0.2\times \left(R_{700}-R_{550}\right)\times \left(R_{700}/R_{670}\right)\right]; \end{array}$$

Optimized Soil-Adjusted Vegetation Index (OSAVI) (Rondeaux et al., 1996),

$$\begin{array}{l} OSAVI=\left(1+0.16\right)\times \left(R_{780}-R_{670}\right)/\left(R_{780}+R_{670}+0.16\right); \end{array}$$

and the TCARI/OSAVI ratio (Rondeaux et al., 1996; Daughtry et al., 2000).

### Statistical analysis

Agronomical traits and VIs were evaluated using multivariate (PCA, principal component analysis) and univariate (ANOVA, analysis of variance) analyses with the programs CANOCO 4.5 (Microcomputer Power, Ithaca NY, USA) and SPSS 22.0 (IBM Corp., Armonk, NY, USA). ANOVA was conducted to calculate differences between genotypes (Meseta, Jallon and Smooth), nitrogen treatments (see Table 1) and their interaction. When there were differences between treatments means, they were assessed using Tukey's HSD test. The results were accepted as significant at *P* < 0.05. Most of the traits were not significantly altered by the interaction genotype × N treatment. Therefore, we presented in this study the significant effects for the main factors independently. A correlation matrix was generated in R environment for evaluating the linear relationships between all parameters analyzed. Visualization of significant correlations was performed using the software Cytoscape 3.4.0 (Shannon et al., 2003). The significance threshold for correlations between traits was set at *r* > 0.6 for positive correlations and *r* < −0.6 for negative correlations, with a *P*-value < 0.001 in both cases. The figures for the PCA were built in CanoDraw 4.0 (Microcomputer Power) and for agronomical traits and linear regressions in SigmaPlot 11.0 (Sysat Software Inc., Point Richmond, CA, USA). Stepwise regressions between grain yield and VIs were performed in SPSS 22.0 to develop prediction models for grain yield. The proportion of variance explained by each predictor was calculated in R environment using the package *relaimpo*.

## Results

Nitrogen use indexes as presented in Table 2 include the agronomical nitrogen use efficiency (aNUE) and N partial factor productivity (NPFP) according to the 10 N application regimens as detailed in Table 1 for the two hybrid (Jallon and Smooth) and one conventional genotype (Meseta) of the study. While the aNUE is calculated in reference to the N0 regimen yield in order to estimate the use efficiency of the fertilizer applied on top of the residual nitrogen found present in the soil, NPFP adjusts for yield in reference to the applied nitrogen supply per treatment without reference to the other treatments. There were no significant differences found between the three genotypes in the aNUE calculations, there are several clear separations in NPFP between the hybrid and conventional genotypes, notably at N130a, N150b, N170c, and N170d.

**Table 2:** Agronomical nitrogen use efficiency (NUE) and N partial factor productivity according to the 10 N application regimens as detailed in Table 1.

	**Agronomical NUE (g grain g N**^**−1**^**)**				**N partial factor productivity (g grain g N**^**−1**^**)**			
	**Meseta**	**Jallon**	**Smooth**	***P***	**Meseta**	**Jallon**	**Smooth**	***P***
N130a	20.12	13.89	22.38	0.057	**46.93 a**	**46.69 a**	**57.06 b**	**0.017**
N130b	21.69	19.13	20.91	0.857	48.51	51.94	55.58	0.376
N150a	18.09	16.67	17.65	0.946	41.34	45.10	47.69	0.402
N150b	17.75	20.44	20.05	0.381	**40.99 a**	**48.87 b**	**50.10 b**	**0.006**
N150c	20.89	21.11	23.00	0.855	44.13	49.54	53.05	0.171
N170a	17.84	11.97	18.24	0.053	**38.35 a**	**37.06 a**	**44.75 b**	**0.028**
N170b	18.90	15.50	16.80	0.479	39.41	40.58	43.31	0.381
N170c	19.51	17.96	19.43	0.420	**40.01 a**	**43.04 ab**	**45.94 b**	**0.009**
N170d	17.28	18.61	23.45	0.128	**37.78 a**	**43.69 ab**	**49.96 b**	**0.011**

*N0 is used for reference in the calculation of each and as such is not shown. Treatments in bold indicates P < 0.05*.

**Figure 2:**
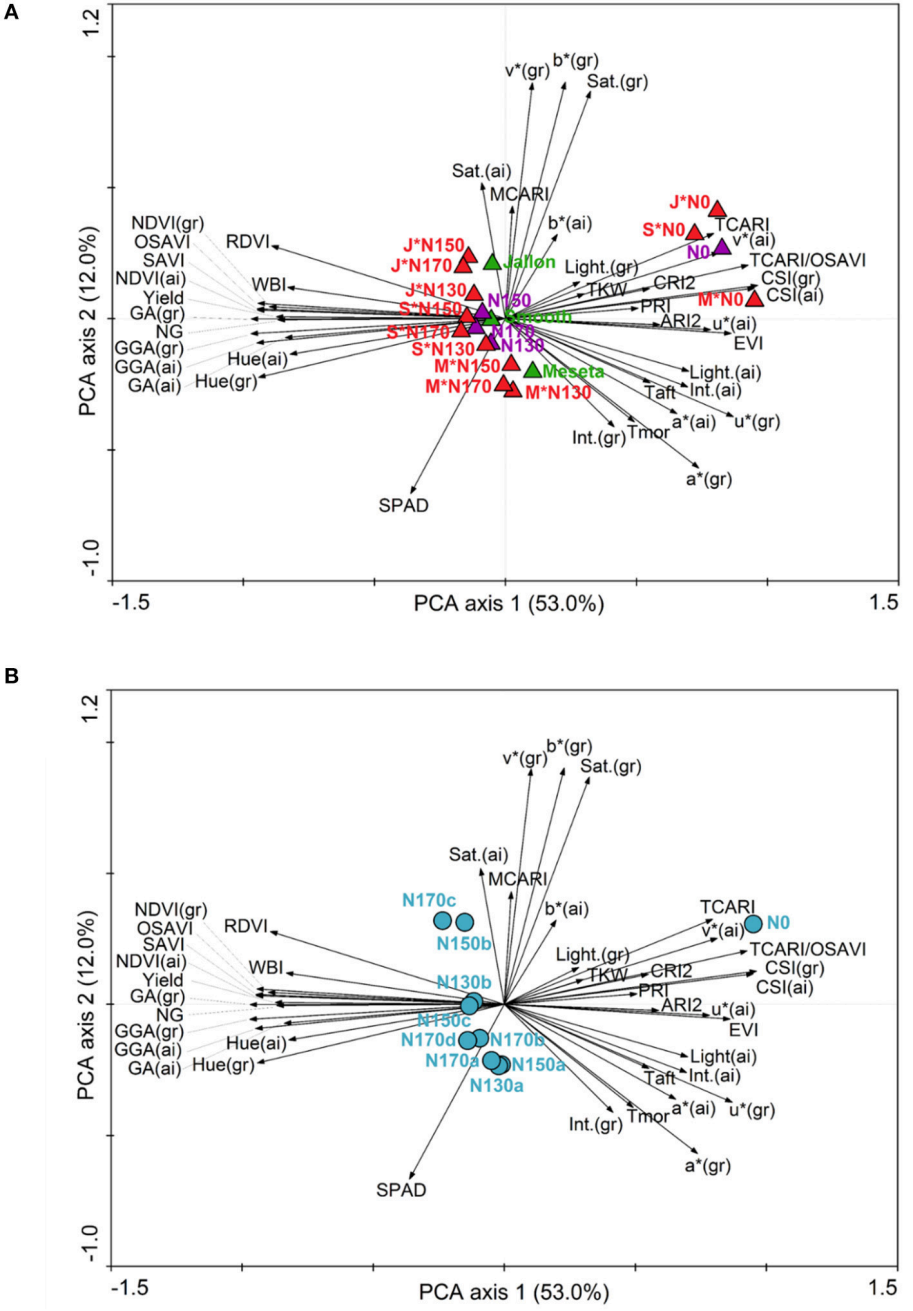
Principal component analysis (PCA) of agronomical and physiological traits in three barley genotypes at different N levels. Arrows represent the variables and triangles the different genotypes (green), N levels (purple) and their interaction (red) in **(A)**, and circles the different N levels and application dates in **(B)** according to Table 1. M, Meseta; J, Jallon; S, Smooth.

A close inspection of the principal component analysis (PCA) combining the agronomical and physiological traits of the different levels and application regimens of the study trial design and the measured parameters from the UAV HTPP and field data (Figure 2) demonstrates which field methods have measured similar crop attributes and how they are related to the different genotypes and N levels (Figure 2A) and to the different N levels and application dates (Figure 2B). In both PCA's there is a clear separation on PCA axis 1 between variables associated with higher total green biomass (GA, GGA, Hue, NDVI, OSAVI) and pigment/stress (CSI, v^*^, TCARI/OSAVI, PRI, ARI2). The second PCA axis appears to have separated SPAD as a leaf measurement from some of the alternatives to RGB color space that are often considered as indicators ground cover (Saturation, v^*^ and b^*^). In Figure 2A it appears that the lower nitrogen application levels positioned more associated with the pigment/stress plant field measurements while the higher nitrogen applications and yield are on the side of higher total green biomass. Meseta, the conventional variety, appears opposite measures of total ground cover and green biomass. In Figure 2B, again N0 is associated strongly with pigment/stress, while the rest are only slightly on the side of total biomass and more spread along the PCA axis 2, indicating differences in chlorophyll leaf concentration (SPAD) and percent ground cover. There is little difference noted between the different nitrogen application levels (130–170 kg ha^−1^), with more separation between the application timings (a, b, c, d), with the largest separation between N170a and N170c.

**Figure 3:**
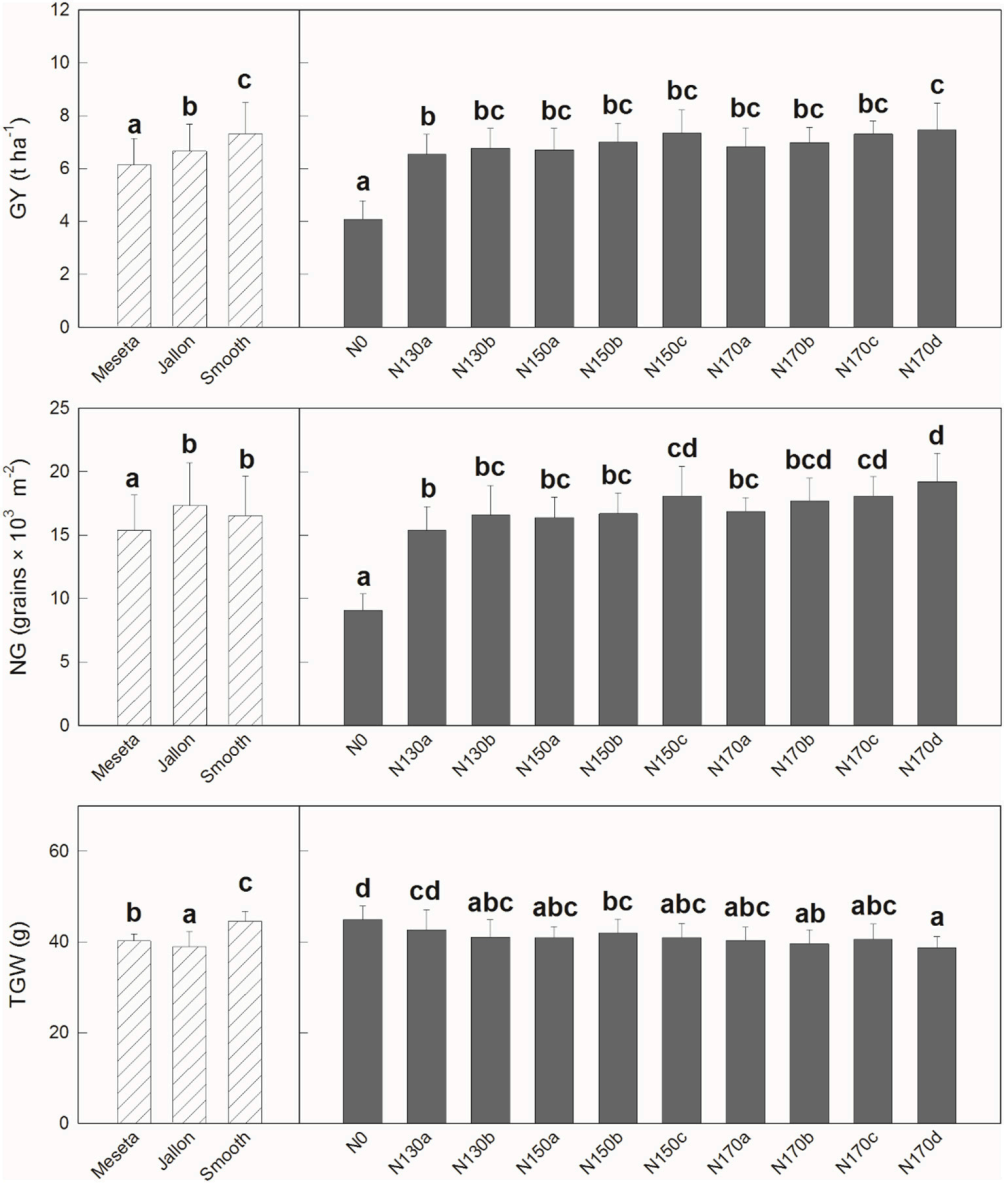
Grain yield (GY), thousand grain weight (TGW), and number of grains per area (NG). Each value is the mean ± *SD* for each genotype and nitrogen supply (*n* = 30 for genotypes and *n* = 9 for N supplies). Bars with different letters are significantly different at *P* < 0.05.

**Figure 4:**
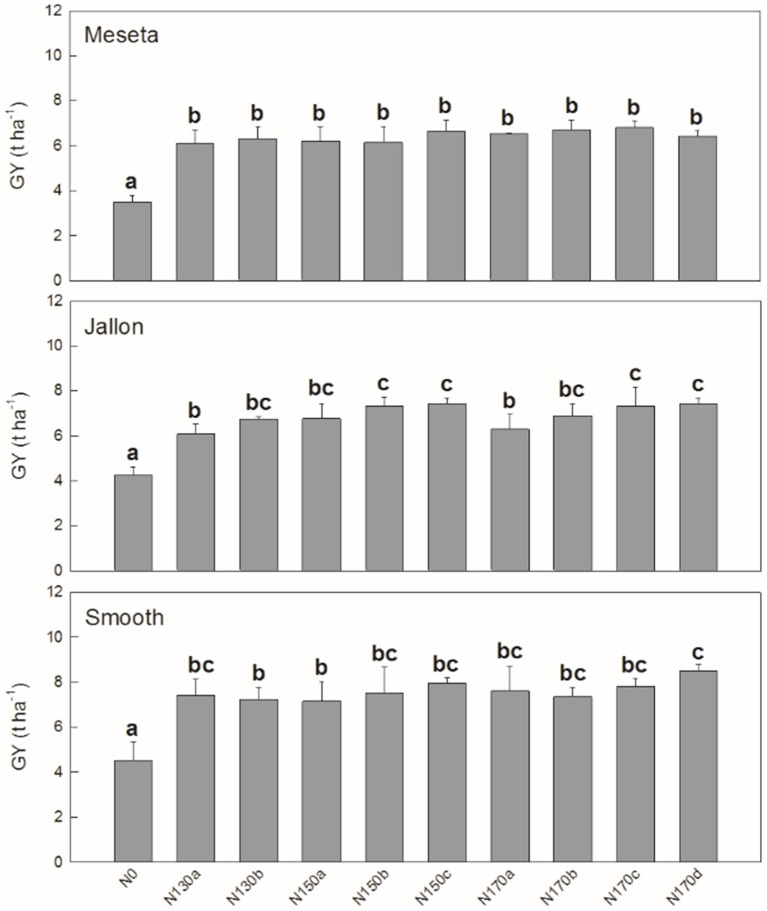
Meseta (conventional), Jallon (hybrid), and Smooth (hybrid) barley varieties. Each value is the mean ± *SD* for each genotype separately for each nitrogen supply (*n* = 3 for genotype replicates and *n* = 9 for N supplies). Bars with different letters are significantly different at *P* < 0.05.

Similar patterns are observed when comparing the different varieties in the agronomical components in Figure 3. We see significant differences in grain yield (GY) and thousand grain weight (TGW) between the three varieties, but in terms of number of grains (NG) the hybrids Smooth and Jallon are together higher in comparison to the lower NG of the Meseta. In nearly all cases, not surprisingly N0 is significant lower, but in terms of GY, only N130a and N170d show significantly different effects, while there is only slightly more separation in terms of the yield subcomponents into a total of four groups for NG and TGW. In all three comparisons, there were no separation between N130b and N170b, for example. In Figure 4, looking in greater detail at GY, we observe no interactions between genotype and the non-zero nitrogen application regimens. There were no differences at all in the conventional variety Meseta, and only two slight separations in treatments in the hybrid varieties Jallon and Smooth and more so due to fertilization regimen timing rather than quantity of fertilizer.

**Figure 5:**
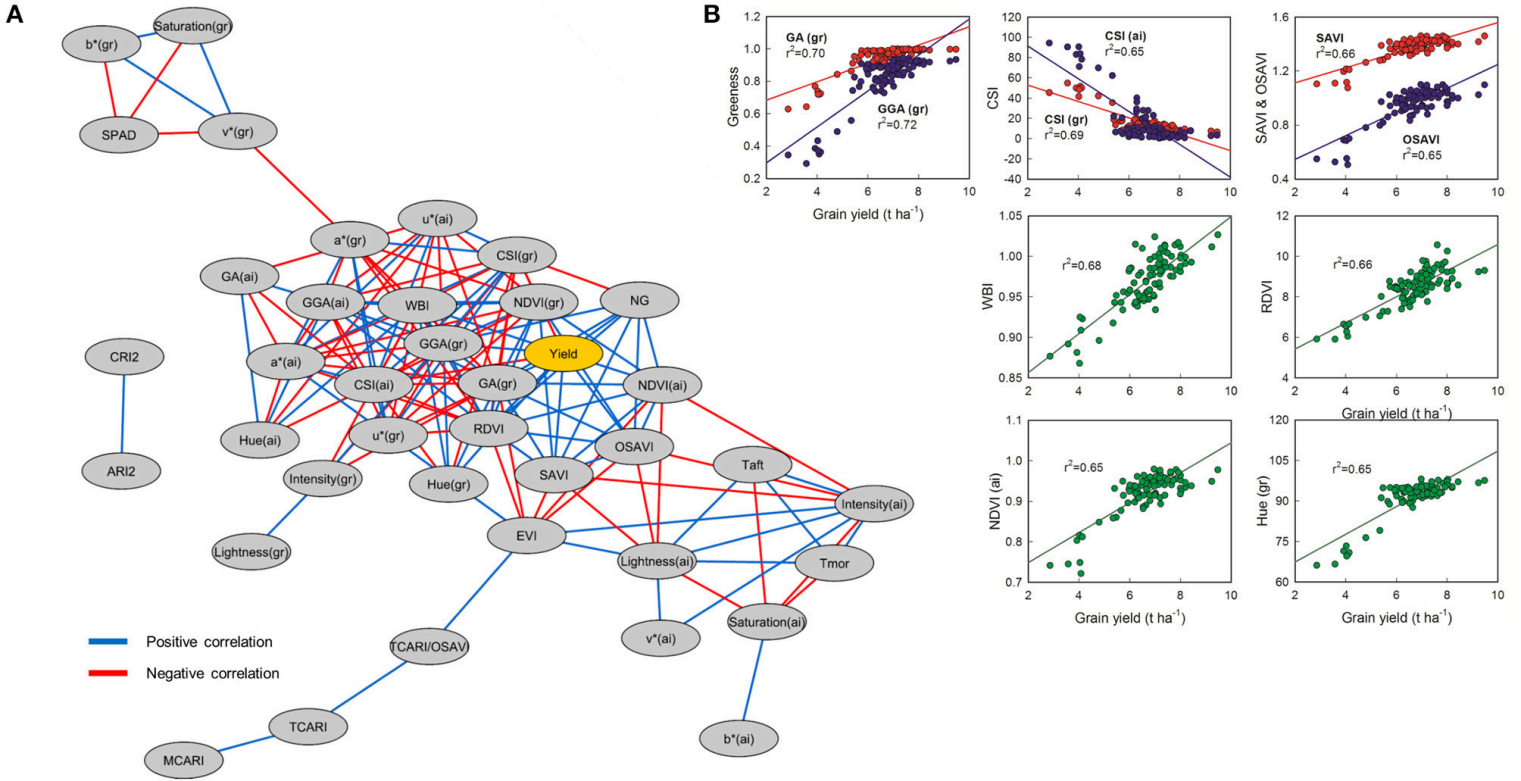
**(A)** Correlation network for physiological traits in barley using three different genotypes and ten nitrogen treatments. Edge color represent positive correlations between traits in blue (Pearson's *r* > 0.6; *P* < 0.001) and negative correlations in red (Pearson's *r* < −0.6; *P* < 0.001). All the significant correlations between yield and other traits are shown in **(B)** (*n* = 90).

The full summary of the different non-destructive ground and aerial VIs presented in Table 3 demonstrates their similar capacity for quantifying the interactions between genotype and treatment. With regards to total N and fertilizer application timing, the physiologically based VIs are frequently able to separate genotypes or differentiate between the conventional and hybrid varieties. We also observe similar results from aerial and ground measurements, as with the high resolution RGB image analyses. However, little consistency is observed between the non-zero N application quantities, with somewhat more distinction made between treatments different in both nitrogen quantity and timing. For example, there were significant differences between the minimal number of applications in N130a and N150a regimens and the three to four field applications in N170c and N170d treatments (NDVI, Hue, and GGA from the ground measurements; and additionally NDVI, RDVI, SAVI and OSAVI from the aerial multispectral data). All of these physiological indices are considered indicators of total green biomass, albeit from different sensors and spectral regions. The capacity for these total green biomass physiological indices to track differences in yield is further corroborated in the yield correlation network presented in Figure 5A, where yield is effectively surrounded by the same set of VIs that were capable of detecting differences related to the combination of N amount and timing (GA, GGA, NDVI, RDVI, SAVI, and OSAVI).

**Table 3:** Vegetation indices from ground and from aerial images and canopy temperature (*n* = 30 for genotypes and *n* = 9 for N supplies).

	**Genotype**			**N supply**									
**Trait**	**Meseta**	**Jallon**	**Smooth**	**N0**	**N130a**	**N130b**	**N150a**	**N150b**	**N150c**	**N170a**	**N170b**	**N170c**	**N170d**
**VEGETATION INDICES FROM GROUND**													
NDVI	0.67 a	0.71 b	0.73 b	0.50 a	0.70 b	0.72 bc	0.71 bc	0.75 cd	0.71 bc	0.73 bc	0.72 bc	0.77 d	0.72 bc
SPAD	44.3 b	41.1 a	43.6 b	36.1 a	45.8 b	44.1 b	45.1 b	38.2 a	45.2 b	47.0 b	44.8 b	38.9 a	44.5 b
Intensity	0.334 b	0.311 a	0.310 a	0.327	0.326	0.316	0.318	0.312	0.317	0.319	0.315	0.313	0.319
Hue	86.6 a	91.6 b	93.4 c	71.4 a	94.6 b	93.7 b	93.1 b	92.3 b	93.3 b	94.0 b	93.9 b	93.9 b	95.1 b
Saturation	0.257 a	0.326 c	0.290 b	0.385 c	0.256 a	0.287 ab	0.263 a	0.325 b	0.281 ab	0.263 a	0.283 ab	0.317 b	0.250 a
Lightness	44.0 c	43.2 b	42.3 a	44.0	43.6	43.1	42.6	43.3	43.0	42.8	42.9	43.6	42.7
a^*^	−19.5 c	−23.4 a	−22.1 b	−16.7 d	−21.5 bc	−22.6 abc	−20.9 c	−23.9 ab	−22.0 abc	−21.3 c	−22.3 abc	−24.3 a	−21.0 c
b^*^	27.3 a	31.5 c	29.0 b	33.9 c	27.5 a	29.2 ab	27.3 a	31.6 bc	28.7 ab	27.5 a	28.8 ab	31.4 bc	26.7 a
u^*^	−13.9 b	−17.7 a	−16.7 a	−8.5 d	−16.4 abc	−17.3 abc	−15.6 c	−18.3 ab	−16.7 abc	−16.1 bc	−17.0 abc	−18.9 a	−16.0 bc
v^*^	32.0 a	35.9 c	33.3 b	36.8 c	32.5 a	33.9 abc	31.7 a	36.1 bc	33.3 ab	32.2 a	33.3 ab	36.1 bc	31.4 a
GA	0.916 a	0.965 b	0.966 b	0.737 a	0.966 b	0.970 b	0.959 b	0.985 b	0.968 b	0.968 b	0.974 b	0.996 b	0.970 b
GGA	0.760 a	0.838 b	0.854 b	0.398 a	0.854 b	0.853 b	0.835 b	0.870 bc	0.854 b	0.858 bc	0.865 bc	0.915 c	0.873 bc
CSI	18.0 b	12.4 a	13.9 a	46.4 c	11.6 ab	11.7 ab	13.1 b	11.7 ab	11.9 ab	11.5 ab	11.2 ab	8.1 a	10.2 ab
**VEGETATION INDICES FROM AERIAL IMAGES**													
NDVI	0.912 a	0.928 b	0.927 b	0.788 a	0.915 b	0.940 bcd	0.917 bc	0.957 cd	0.945 bcd	0.927 bcd	0.927 bcd	0.962 d	0.944 bcd
Intensity	0.255 b	0.196 a	0.313 c	0.285 b	0.259 ab	0.270 ab	0.249 ab	0.264 ab	0.213 a	0.237 ab	0.265 ab	0.281 ab	0.224 ab
Hue	91.4 a	88.3 a	98.7 b	83.7	86.7	91.0	95.9	95.2	97.8	97.0	88.9	95.0	88.9
Saturation	0.263 b	0.378 c	0.185 a	0.245 a	0.271 a	0.277 ab	0.277 ab	0.277 ab	0.335 b	0.290 ab	0.238 a	0.238 a	0.297 ab
Lightness	32.5 b	26.2 a	38.6 c	35.3	32.3	34.1	32.3	34.0	28.1	30.8	33.7	35.3	28.5
a^*^	−14.3 b	−14.0 b	−15.3 a	−11.9 c	−12.5 c	−14.4 ab	−15.9 a	−16.0 a	−15.9 a	−15.6 a	−15.2 a	−15.0 a	−12.8 bc
b^*^	21.4 a	27.6 b	20.3 a	21.6 a	20.7 a	22.1 ab	22.1 ab	22.6 ab	29.7 b	24.4 ab	21.0 a	21.3 a	25.9 ab
u^*^	−8.5 b	−7.7 b	−10.4 a	−5.7 b	−6.7 ab	−8.5 ab	−10.3 a	−10.3 a	−10.2 a	−10.0 a	−10.0 a	−9.6 a	−7.3 ab
v^*^	22.8 ab	21.0 a	23.8 b	23.3	21.6	23.6	23.4	24.3	20.7	22.2	22.9	23.5	19.8
GA	0.943	0.944	0.964	0.874	0.938	0.948	0.961	0.962	0.977	0.985	0.948	0.955	0.956
GGA	0.807	0.795	0.861	0.646	0.715	0.796	0.801	0.846	0.864	0.865	0.865	0.870	0.941
CSI	16.5	17.0	12.3	4.5	9.6	11.1	11.4	11.9	12.1	16.3	17.8	26.1	31.7
PRI	−0.107	−0.130	−0.105	0.128 b	−0.129 a	−0.168 a	−0.117 a	−0.203 a	−0.161 a	−0.046 a	−0.069 a	−0.201 a	−0.174 a
SAVI	1.36 a	1.38 b	1.38 b	1.17 a	1.36 b	1.40 bc	1.37 b	1.43 c	1.41 bc	1.38 bc	1.38 bc	1.44 c	1.41 bc
MCARI	27.4	32.4	28.3	34.8	25.0	27.5	25.2	35.3	26.9	28.1	24.4	36.0	30.6
WBI	0.943 a	0.975 b	0.991 c	0.899 a	0.970 bc	0.979 bc	0.974 bc	0.981 bc	0.983 bc	0.965 b	0.981 bc	0.986 c	0.978 bc
RDVI	7.99 a	8.71 b	8.66 b	6.44 a	8.17 b	8.63 bc	8.23 b	9.17 cd	8.51 b	8.47 b	8.62 bc	9.66 d	8.62 bc
EVI	5.02	4.29	4.63	9.84 b	4.91 a	3.67 a	4.84 a	3.31 a	3.52 a	4.78 a	4.61 a	3.28 a	3.68 a
ARI2	−1.40	−1.70	−1.57	−0.09 b	−1.77 a	−1.58 a	−1.48 a	−1.63 a	−1.62 a	−1.67 a	−2.37 a	−2.12 a	−1.24 a
CRI2	−0.019	−0.020	−0.018	−0.001 b	−0.023 a	−0.019 a	−0.019 a	−0.018 a	−0.021 a	−0.021 a	−0.029 a	−0.022 a	−0.016 a
TCARI	22.8 a	24.8 b	23.3 ab	37.3 b	22.8 a	20.5 a	23.2 a	22.8 a	20.6 a	23.8 a	22.5 a	22.3 a	20.4 a
OSAVI	0.930 a	0.975 b	0.971 b	0.642 a	0.940 b	1.004 bc	0.940 a	1.044 c	1.015 bc	0.967 bc	0.965 bc	1.057 c	1.013 bc
TCARI / OSAVI	26.1	27.0	25.2	60.0 b	24.4 a	20.5 a	24.7 a	21.8 a	20.3 a	24.6 a	23.5 a	21.1 a	20.2 a
T_mor_ (°C)	17.3	17.3	17.9	20.2 b	18.9 ab	16.3 ab	18.5 ab	15.7 a	16.5 ab	18.4 ab	18.5 ab	15.6 a	16.3 ab
T_aft_ (°C)	11.7	11.6	12.3	15.3 b	11.9 ab	11.6 a	11.9 ab	10.8 a	11.1 a	11.4 a	11.8 ab	11.1 a	11.8 ab

*Letters are added for each value when differences for genotype and/or N supply are significant at P < 0.05*.

NG is also very closely correlated with GY, while TGW is not. In Figure 5B, the detailed graphs of the VIs most significantly correlated directly with GY are shown, including ground calculations of GA and GGA together (*r*^2^ 0.70 and 0.72, respectively), ground and aerial based calculations of CSI together (*r*^2^ 0.69 and 0.65, respectively), SAVI and its optimized variant OSAVI together (*r*^2^ 0.66 and 0.65, respectively), and WBI (*r*^2^ 0.68), RDVI (*r*^2^ 0.66), NDVI (*r*^2^ 0.65), and Hue (r^2^ 0.65) separately below. The asymptotic effects of signal saturation are observable more strongly in the correlation graphs of both NDVI and Hue, while the WBI and RDVI both appear to hold fairly linear in comparison though linear regressions were used in all cases for the sake of comparison. Morning (T_mor_) and afternoon (T_aft_) temperature were both significantly higher for the N0 treatment compared to N150b and N170c, while only T_aft_ provided additional separation between N0 and N130b, N150c and N170a.

In Table 4 we present multivariate linear models for estimating grain yield using different selections of non-destructive VIs as indicated using both forward and backward stepwise selection techniques, with a standard AIC selection criterion. We also present the proportion of variance explained by each model predictor, in terms of total variance explained by each predictor (sum equaling the total model r^2^) and the standard error of prediction (SEP). All three models presented were found to be significant at the *P* < 0.001 level. Using the UAV platform for image acquisition, which allows for the use of the 11+ILS sensor multispectral camera as well as the same sensors used on the ground, explains a total of 77.8% of the variation in yield for all 90 plots across genotypes under different N supply regimens (total plus application timing) can be explained by the WBI and SAVI indices. In the case of ground based VIs, 71.6% of the yield may be explained by the GGA index alone. In the case of combining VIs from both aerial and ground measurements, a total of 82.7% of yield variation may be explained. In the case of the combined aerial and ground measurements, the RGB indices Hue and GGA taken from the ground level contributed nearly 50% of the final *r*^2^-value to the multispectral VIs WBI and RDVI, with morning temperature making a minor contribution.

**Table 4:** Multivariate regression models explaining grain yield variation from vegetation indices (VIs) across genotypes under different N supplies.

**Predicted parameter**	**Traits**	**Multivariate model**	***r*^2^**	**SEP**	***P***
Grain yield	Vegetation indices (from aerial images)	GY = 17.32 × WBI + 6.49 × SAVI – 19.02	0.778	0.555	<0.001
		Proportion of variance explained by each predictor: - WBI - SAVI	0.402 0.376		
Grain yield	Vegetation indices (from ground)	GY = 1.41 + 6.46 × GGA(gr)	0.716	0.625	< 0.001
		Proportion of variance explained by each predictor: - GGA(gr)	0.716		
Grain yield	Vegetation indices (all)	GY = 15.83 × WBI + 0.17 × Hue(gr) − 7.39 × GGA(gr) + 0.51 × RDVI – 0.48 × T_mor_ – 21.90	0.827	0.499	<0.001
		Proportion of variance explained by each predictor: - WBI - Hue(gr) - GGA(gr) - RDVI - T_mor_	0.210 0.197 0.192 0.181 0.047		

*SEP, standard error of prediction*.

## Discussion

### Total N and N application regimen timing contributions to yield

Directed plant phenotyping efforts toward improving barley nitrogen use efficiency (NUE) and efficacy must take into account the physiological mechanisms that affect NUE, but also consider the associated economic costs of different fertilizer application regimens, namely via plant nitrogen uptake and storage capacity (Raun and Johnson, 1999; Hirel et al., 2007; Anbessa and Juskiw, 2012; Krapp, 2015; Kostadinova et al., 2016; Rajala et al., 2017). Not only the amount of fertilizer applied, but also the number and timing of these applications may contribute to specific growth stages of the crop and thus result in less or greater contribution to the final yield of the crop. In the two approximations of NUE presented in Table 2, the agronomical nitrogen use efficiency (aNUE) and N partial factor productivity (NPFP), we may observe some potential for error in each as an approximation of actual NUE in the case of not fully accounting for residual soil nitrogen. While there were no differences in aNUE between the hybrids (Jallon and Smooth) and the conventional genotype (Meseta), we are also able to note that the yield from both the hybrids was quite a bit higher than the conventional variety in the N0 treatment. This in of itself may be interpreted as an indication of higher NUE by the hybrids at low N levels, something in of itself of potential interest. Also, since N0 is the reference point for the other N treatments' aNUE, the higher yield of the hybrids already at N0 becomes a strong weighing factor in subsequent calculations. The separations in NPFP between the hybrid and conventional genotypes, notably at N130a, N150b, N170c, and N170d, if they may be assumed as better indicators of relative NUE between the genotypes, again serves to highlight the importance of management practice on NUE, potentially indicating more strongly the different capacities of each genotype for nitrogen uptake and storage.

Too minimal number of field applications will result in an over application of fertilizer at any one time, exceeding the crop uptake capacity and result in loss of excess fertilizer due to leaching during rain or irrigation. Similarly, temporal infrequency with long time periods between fertilizer applications may exceed the plant capacity for N storage and stunt growth in subsequent stages or trigger N mobilization in a way that lowers yield by reducing total photosynthetic biomass prior to grain filling. These concepts are supported by the first comparisons of the data presented in this study where indeed the only differences observed between the different non-zero nitrogen regimens are in terms of the application frequency rather than the total fertilizer application amounts. There is more separation in the PCAs presented in Figure 2B where fertilizer timing by genotype is considered compared to Figure 2A where only the total amount is considered. This is even more clearly supported by Figure 3 where the only differences in GY, NG, and RGW were found between the N130a regimen and the N170c and/or N170d regimens, indicating that the increase in total N is only to be marginally effective if also increasing the frequency of application. In no instance was there any distinction between N130a and N170a, suggesting an exceedance of N uptake and/or storage capacity in terms of yield production.

In Figure 4, with the detailed look at the full fertilizer regimen by genotype, there is no separation at all between non-zero N treatments in the conventional variety Meseta. The lowest fertilization rate (130 kg ha^−1^) applied in both two or three doses were sufficient to exceed the nitrogen uptake/assimilation capacity of Meseta. Therefore, tilling costs could be reduced with the application of less amounts and frequency of nitrogen fertilizer. Jallon appears to have a greater capacity for fertilizer uptake and use as evidenced by increased yield at higher fertilizer applications with increased frequency. The analysis of the application dates suggested that initial nitrogen fertilization during pre-sowing guarantee higher grain yields at 130, 150, and 170 kg ha^−1^. This could be an advantage due to better crop establishment and initial tiller development (Baethgen et al., 1995). The lesser degree of separation between the different frequencies of application (a, b, c, d) in Smooth compared Jallon may suggest a greater capacity for N storage for subsequent mobilization at critical yield contributing growth stages. For instance, average GY in Smooth was higher than in Jallon. Overall, in both barley hybrids there is a tendency to higher GY when nitrogen is applied at sowing. It may indicate that the contribution of preanthesis reserves to grain filling is crucial (Van Sanford and MacKown, 1987; Baethgen et al., 1995). These findings suggest that the rate and timing of nitrogen application depend on each genotype. Therefore, the characterization of the different nitrogen strategies in barley genotypes could be relevant to significantly reduce the costs of fertilizers and tilling and the potential ground water contamination by nitrogen leaching.

### Non-destructive vegetation indices for ground and UAV phenotyping

Genotypic differences in VIs between the conventional line, Meseta, and the hybrids, Jallon and Smooth, suggest that the hybrids presented greater canopy biomass and green area, water status and delayed senescence (Table 3). These traits were correlated with GY, as indicated in the correlation network (Figure 5), which suggest that the greater canopy biomass, water status and the delayed senescence in the hybrids, especially in Smooth, were an advantage over the conventional line. The hybrids showed greater capacity for fertilizer uptake with higher GY when nitrogen is applied at sowing, as we reported above, which could have led to a higher tillering and crop canopy cover during vegetative growth as VIs indicated. In previous studies, a rapid development of wheat plants was considered a positive trait for plant performance and to avoid abiotic stresses (Bort et al., 2014; Medina et al., 2016).

From a methodological perspective on the use of UAVs for phenotyping, especially in terms of non-destructive measurements for the selection of different levels of performance by phenotype and treatment, both the high resolution RGB and the multispectral VIs presented here performed comparatively similar. Both class of VIs here managed to track the different levels of performance from each of the three barley varieties in terms of final post-harvest yield (Table 3) equally well as in the comparison of the final yield and yield parameters themselves (GY, NG, TGW; Figures 3, 4). This presents a case for the potential benefits of this technology for modernizing traditional plant phenotyping programs for both the improvement of throughput in terms of time and labor spent in the field as well as the amount of time that the crop must be grown before selection is possible, both of which also represent cost savings (Fiorani and Schurr, 2013; Araus and Cairns, 2014; Hawkesford and Lorence, 2017). Furthermore, the comparable performance of the relatively low cost RGB sensors, with use of appropriate methodology as applied in this study, present a viable alternative to the use of advanced scientific instruments such as the Tetracam MCA11+ILS for plant phenotyping trials and studies (Kefauver et al., 2015; Vergara-Diaz et al., 2015; Zhou et al., 2015).

Still, the use of UAVs also enables the deployment of these image analysis techniques for calculating various VIs to the realm of field phenotyping with both improved throughput (capability to cover hectares in minutes) and complete individual plot coverage of whole trials to provide a more complete capture of variability in field conditions compared to other point or subset area field measurements such as SPAD or Greenseeker NDVI (Fiorani and Schurr, 2013; Araus and Cairns, 2014; Zaman-Allah et al., 2015). On the other hand, more advanced sensor technology, such as field spectroscopy or hyperspectral imaging sensors, may offer some improvements in N content estimation and other relevant physiological parameters (Clevers and Kooistra, 2012; Zarco-Tejada et al., 2012; Pölönen et al., 2013; Bareth et al., 2015; Gonzalez-Dugo et al., 2015).

### Contributions of UAV plant phenotyping platform for improving barley NUE and application regimen efficacy

The highest correlations with final grain yield came from the GGA and GA indices from RGB images taken at the ground level followed closely by the same indices measured from the UAV aerial platform. In the application of multivariate models and stepwise selection as presented in Table 4, GGA alone was only slightly outperformed by two multispectral indices (*r*^2^ 0.716 vs. 0.778). This may be related to the capacity of the multispectral indices to measure plant physiological components separately, such as with the WBI and SAVI indices selected here (Huete, 1988; Gamon et al., 1992; Peñuelas et al., 1993), whereas the RGB indices most likely calculate a combination of physiological components or overall performance related to biomass and/or total green biomass (Casadesús et al., 2007; Casadesús and Villegas, 2014). In this sense, different multispectral indices may be more often complimentary in a multivariate model compared to the quantification provided by high resolution RGB covering only broad electromagnetic regions in the visible spectrum.

As seen in the final multivariate model combining all the VIs from the ground and the UAV, the optimal combination may be found in a selection of the best estimates overall crop performance (RDVI and GGA contributing 0.192 and 0.181 to the total model *r*^2^) and some multispectral index specific to the target traits of the study, such as WBI, Hue or temperature, which may be tracking more specific traits such as pigment quality and root growth, representing traits more specific to the varying nitrogen regimens of this study (contributing 0.210, 0.197, and 0.047 to the total model *r*^2^). Interesting enough the split between more general performance indices and specific indices appears to be about even, though it can be argued that the WBI in a lack of water stress conditions may also be tracking biomass more closely than actual water stress, since it is a measure of total water in the plant canopy and is thus also strongly affected by total plant biomass (Huete, 1988; Huete et al., 2002). Hue can be seen as a potential pigment quantification replacement for the multispectral pigment indices of ARI2, CRI2, MCARI, TCARI, and TCARI/OSVI, but was here found to be much more closely related to yield, which may be due to the fact that at high spatial resolution sampling, it must also contain some component of biomass as it separates out vegetation fractional cover based soil background color separation from plant photosynthetic and non-photosynthetic vegetation.

The relevance of the inclusion of both WBI and mid-morning temperature (T_mor_) may even be interpreted as factors related to increased root growth that allowed for increased nutrient uptake capacities in the higher yielding hybrid varieties. Other previously discussed comparisons in terms of the difference in performance with fertilizer application timing and number of applications may also support this; however, since this study did not include specifically root measurements, that cannot be confirmed here, as it has been in other studies (Postma et al., 2014; Gioia et al., 2015). Still, as UAV and sensor technology and processing continues to advance, we may expect their contributions to high-throughput plant phenotyping to similarly increase (Hruska et al., 2012; Suomalainen et al., 2014; Gevaert et al., 2015).

## Conclusions

Nitrogen management, including rate and timing, is a key factor controlling grain yield in barley genotypes. The selection of hybrids with a better plant performance compared to lines, such as greater crop canopy cover, water status and delayed senescence, could contribute to the enhancement of barley yield stability and nitrogen use efficiency. UAV platforms and associated technology including aerial platform control and stability, appropriate affordable scientific research sensors and processing software capacities have advanced sufficiently to be of use for HTPP studies in field conditions. This technology allows for the development of phenotyping selection criteria for yield under different experimental trial conditions, including but not limited to the nitrogen fertilizer regimen treatments in this study, or for the selection toward improving specific physiological capacities such as nitrogen use efficiency or nitrogen uptake or storage and remobilization capacity.

## Author contributions

SCK led the writing of the manuscript and coordinated the research project and RV led the statistical analyses and figure preparation. SCK, RV, JA, and SK contributed to the majority of the critical revision of the manuscript text. SCK, RV, OV, SK, MS, and JA all contributed significantly to the field and UAV data acquisition, data processing, and experimental data interpretation. SK, AL, and JM led the design of the experimental trial, the management of the trial cultivation, and the collection of the agronomical data at harvest. All authors have revised the work for intellectual content and have contributed to and approved the final content.

### Conflict of interest statement

The authors declare that the research was conducted in the absence of any commercial or financial relationships that could be construed as a potential conflict of interest.

## Abbreviations

ARI2: anthocyanin reflectance index 2
CRI2: carotenoid reflectance index 2
CSI: crop senescence index
EVI: enhanced vegetation index
GA: relative green area
GGA: relative greener area
GY: grain yield
HTPP: high throughput plant phenotyping
MCARI: modified chlorophyll absorption ratio index
NDVI: normalized difference vegetation index
NG: number of grains per area
NUE: nitrogen use efficiency
OSAVI: optimized soil-adjusted vegetation index
PRI: photochemical reflectance index
RDVI: renormalized difference vegetation index
RGB: red-green-blue
SAVI: soil adjusted vegetation index
TCARI: transformed chlorophyll absorption ratio index
TGW: thousand grain weight
UAV: unmanned aerial vehicle
VIs: vegetation indices
WBI: water band index.
